# Weber’s Experiments on the Vagus Nerve

**Published:** 1846-05

**Authors:** 


*Weber's Experiments on the Vagus Nerve.*—With the aid of a powerful electro-galvanic machine, when the medulla oblongata of a frog, or the ends of the vagi divided at their origin were irritated, the heart was immediately deprived of motion; when the irritation was discontinued, it recommenced beating after a short interval of time—slowly at first, then quicker, and at length its motions returned as before the experiment. When the rotation of the machine is not very rapid, the heart's movements are only retarded and rendered feeble. Tetanic contractions of the heart have never been observed when its motions had been stopped in the manner indicated, but the organ is flattened and its fibres relaxed. Irritation of the vagus of one side only, produces no change in the heart's motion. If the irritation of the vagi is continued sufficiently long to exhaust their excitability, the heart recommences its beating. The parts in the vicinity of the heart connected with the filaments of the sympathetic nerve being irritated in this manner neither retard nor suspend the motions of the organ; on the contrary, they seem to become more frequent, and if the motions of the heart had been previously stopped, they are restored by this last proceeding. It is uncertain if, in this case, this phenomenon should be attributed to irritation of the sympathetic, or if it does not rather depend on the electricity having been transmitted directly to the heart by means of the moist surrounding animal tissues. If the wires of a galvano-magnetic apparatus be applied directly to the heart, the heart may be seized with a tetanic contraction, and its motions be arrested during the continuance of this spasm. Irritation of the vagi in the manner above mentioned produces the same effects in rabbits as in frogs.—[The results of M. Weber's observations are strongly in favour of the opinions of those who maintain that the heart's action is under the influence of the vagi.]—*Ibid.*

